# Growing up in the Betsileo landscape: Children’s wild edible plants knowledge in Madagascar

**DOI:** 10.1371/journal.pone.0264147

**Published:** 2022-02-17

**Authors:** Vincent Porcher, Stéphanie M. Carrière, Sandrine Gallois, Herizo Randriambanona, Verohanitra M. Rafidison, Victoria Reyes-García

**Affiliations:** 1 Institut de Ciència i Tecnologia Ambientals, Universitat Autònoma deBarcelona, Barcelona, Spain; 2 SENS, IRD, CIRAD, Montpellier, France; 3 Faculty of Archaeology, Leiden University, Leiden, The Netherlands; 4 Centre National de Recherches sur l’Environnement (CNRE), Antananarivo, Madagascar; 5 Faculté des Sciences, Université d’Antananarivo, Antananarivo, Madagascar; 6 Institució Catalana de Recerca i Estudis Avanç ats (ICREA), Barcelona, Spain; 7 Department of Anthropology, Universitat Autònoma de Barcelona, Barcelona, Spain; Universidade Federal de Pernambuco, BRAZIL

## Abstract

Understanding local knowledge about wild edible plants (WEP) is essential for assessing plant services, reducing the risks of knowledge extinction, recognizing the rights of local communities, and improving biodiversity conservation efforts. However, the knowledge of specific groups such as women or children tends to be under-represented in local ecological knowledge (LEK) research. In this study, we explore how knowledge of WEP is distributed across gender and life stages (adults/children) among Betsileo people in the southern highlands of Madagascar. Using data from free listings with 42 adults and 40 children, gender-balanced, we show that knowledge on WEP differs widely across gender and life stage. In addition, we find that children have extended knowledge of WEP while reporting different species than adults. Women’s knowledge specializes in herbaceous species (versus other plant life forms), while men’s knowledge specializes in endemic species (versus native or introduced). Finally, we find that introduced species are more frequently cited by children, while adults cite more endemic species. We discuss the LEK differentiation mechanisms and the implications of acquiring life stage’s knowledge in the highland landscapes of Madagascar. Given our findings, we highlight the importance of considering groups with under-represented knowledge repositories, such as children and women, into future research.

## Introduction

Among the numerous services provided by plants, food provision is probably the most important for humans [1, 2]. For many Indigenous Peoples and local communities (IPLC), local ecological knowledge (LEK) about wild edible plants (WEP) allows maintaining local livelihood and cultural identity [3]. LEK about WEP is also critical for safeguarding biocultural diversity and local resilience in times of food scarcity [4, 5]. LEK, however, is drastically threatened by multiple factors and rapid socio-ecological changes–e.g., habitat lost, or species and language extinction [6–8].

An important finding of previous research on LEK is that this body of knowledge is often unevenly distributed within the same society [9], although not much of this research has focused on WEP knowledge [10]. For example, research shows that intracultural variability in knowledge distribution is influenced by socio-economic characteristics of people, such as gender or life stage [11, 12]. This is so because such characteristics shape people’s involvement in subsistence activities, and therefore interaction with the environment or specific environmental features. Therefore, considering individual characteristics when documenting the knowledge of a society is key to identifying the principal repositories of LEK [13].

Among the factors that shape intracultural distribution of knowledge, much research has focused on gender and age [14–16]. Regarding age, studies explored the intracultural differences due to generational variations [14, 15, 17], but very few have embedded children into the sampling [18]. So far, most research addressing knowledge distribution within a society describes age as a factor shaping knowledge accumulation, rather than knowledge differentiation [15, 19]. In other words, children are generally considered as “adults to be”, rather than a group with distinctive knowledge [20].

Despite this general view, recent work in ethnobiology has highlighted that children display specific knowledge that they do not necessarily share with adults [21], or what authors call « children’s culture » [22–24]. The finding is important because it question assumption that the transmission of cultural knowledge in childhood is mainly vertical, i.e., knowledge being transmitted from adults to children, children being mostly the receivers in the process of knowledge transmission [25]. Moreover, the finding that children have differentiated knowledge also has implications for our understanding of children’s potential contribution to livelihoods activities [26] and food systems [27]. For example, using their specialized knowledge, hunter and gatherer Mikea children from south-west Madagascar gather wild edibles that constitute a substantial part of their daily intake [28]. These gathering activities have important positive impacts on children’s health [29].

In this study, we examine intracultural variability in wild edible plant knowledge distribution among the Betsileo, Madagascar. We focus on Madagascar because while LEK on WEP have been well studied in several megadiverse regions such as Amazonia [30] and New Guinea [31], Madagascar has received less attention, despite being a hotspot of biological and cultural diversity. Madagascar, which counts with 12 indigenous languages [32] and 11.399 native vascular plant species known [33], is particularly touched by land cover shifts [34], climate change impacts [35] and more recently by an extreme food crisis [36]. We focus on differences in knowledge distribution associated to gender and life stage (adults vs children) with a particular focus on children. We also look at gendered differences in knowledge distribution, as previous research shows that gender is a structuring factor in shaping knowledge distribution. By using an intersectional lens, i.e., considering gender and life stage, as knowledge differentiation factors, this study contributes to the advancement of existing literature on gendered LEK and questions assumptions on cultural knowledge transmission and acquisition during childhood [18]. We work with southern Betsileo population because the knowledge of this group has not been largely documented, although a previous study suggested that Betsileo’s children hold large amounts of environmental knowledge [37, 38], making this case study an ideal to test life stage variations in LEK.

## Material and methods

### Study area

**Fig 1:**
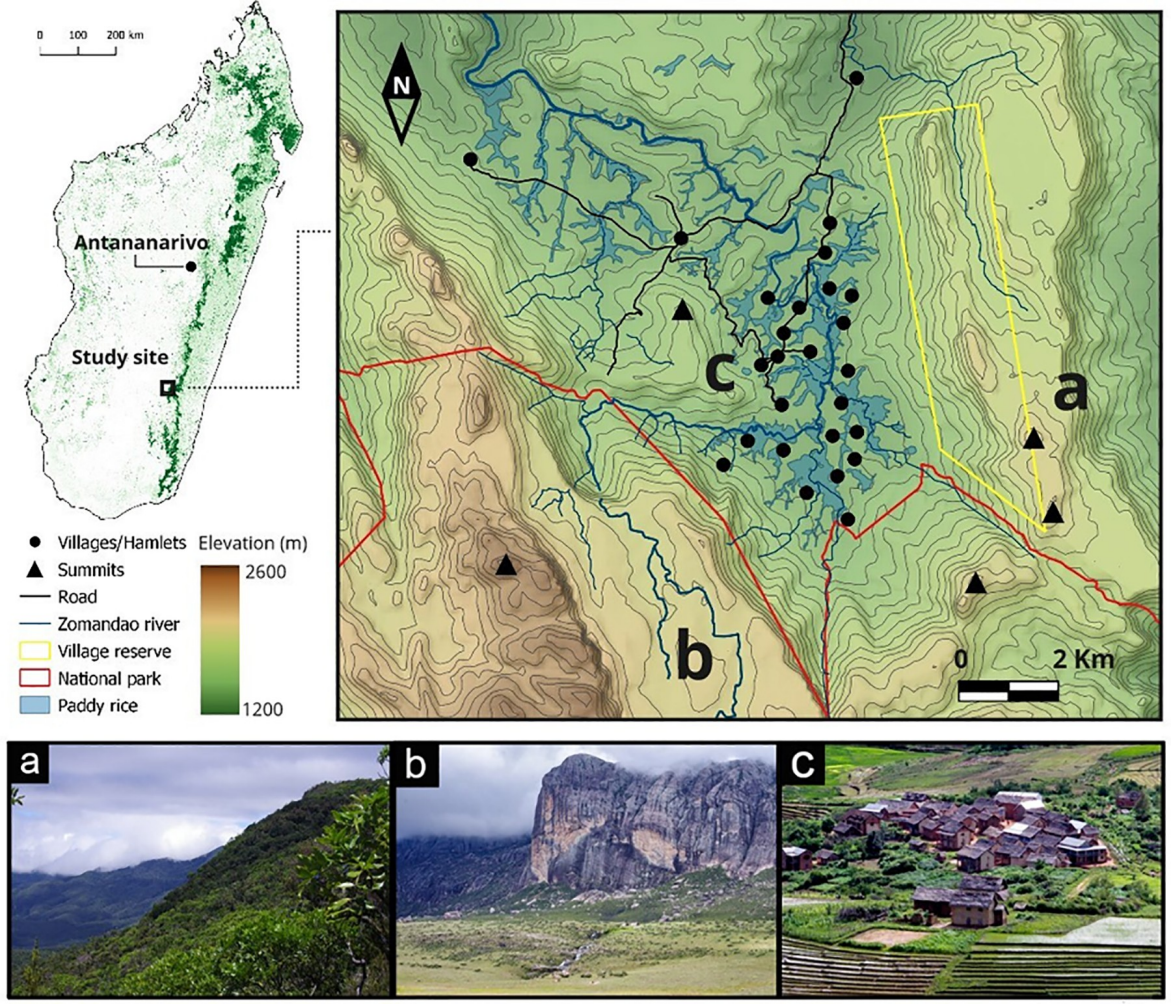
Map of the study site: The Namoly valley landscape and their main ecological zones. **a**. eastern slope: moist altitude dense forest (1500–1800m) and sclerophyllous moist forest (1800–2000), **b.** western slope: altimontane meadow, ericoid thickets (2100m) and rupicolous vegetation (2500m), **c.** valley bottom (1500m): surrounding houses, pastures, crop fields. The map was built under QGIS 3.10.0 by the authors, using elevation SRTM data at 90m spatial resolution from the CGIAR-CSI database [47]. Pictures: V. Porcher. See also Fig 2 for altitudinal zonation of vegetation in Namoly valley.

Fieldwork was carried out in the Namoly valley (-22.12377, 46.92166), southeast of the central highlands of Madagascar (Fig 1). The study area is located on the edge of the Andringitra National Park with high altitudinal gradients (720 to 2658 m). The area has been occupied since the end of the 19th century by the Betsileo people, the third largest ethnic group in the island [39–41]. The topography and the park display a rich ecological habitat mosaic surrounding Betsileo settlements, at the bottom of the valley (1500 m above sea level). On the eastern slope (Fig 1A), moist altitude dense forest (1500–1800m) and sclerophyllous moist forest (1800–2000) support many subsistence economic activities and knowledge associated with forest [42–44]. On the western slope (Fig 1B), the Precambrian granitic massif—a large inselberg culminating at 2658m –supports rupicolous vegetation on its top (2500m) and altimontane meadow and ericoid thickets down the cliff (2100m). This highland vegetation holds the richest botanical diversity and the highest rate of endemism of Madagascar [45, 46]. At the bottom of the valley (Fig 1C), the landscape is made up of a mosaic of rice field and secondary regrowth vegetation that is both woody and shrubby, mainly open and colonized by ferns, surrounding the houses, pastures and other crops fields. Given its location, Betsileo people from the Namoly valley have access to a large diversity of resources and, particularly, to a large choice of wild edible plants. The valley counts approximately 3.800 inhabitants distributed in 662 households isolated or clustered in *vala* (hamlets) of 3 to 15 houses. The isolated houses and hamlets are spread all along the valley, distant from each other by about 500m.

### Betsileo social organization

Up to this date, the Betsileo have a complex system of social stratification structured by patrilineal kinship lines, where male elders play a central social role [41, 48]. The social organization and traditional rules maintained by the traditional chefs (*Ray aman-dreny*) lead to a division of labor by age and gender. This differentiation is particularly marked in subsistence activities [49]. Betsileo people consider children, *kilonga*, individuals between 4 and 14 years of age (before they are *zaza kely*, infant). Betsileo consider that after childhood, individuals have an extended period of transition to adulthood, which might last from 14 to 40 years of age, during which individuals are named *tanora*. Around 18 years old, Betsileo people have their first wedding and create a new household. People consider that a person reach adulthood, *olon-dehibe*, by the age of 40 years. People become elders, *antitra*, after 60 years of age. For the purpose of the study, we will define children as individuals under 18 years old [50].

As many other Malagasy ethnic groups, the Betsileo have adopted an agro-pastoral livelihood system based on a combination of irrigated lowland and rainfed rice cultivation and zebu breeding. All household members work together during the rice harvesting season (*asotry*). However, men take care of zebus and prepare the rice fields. Men also engage in activities that require mobility, i.e., gathering, fishing, hunting, exchange and trade of goods, and take the zebus to specific pastures during the year. All these activities expose them to different ecological habitats across the valley. In turn, women take care of the rice nurseries and work in home gardens and other crops. Women’s activities are mostly organized around the settlements and cover less environments than men’s activities. Children are often raised in an alloparental care system involving several households from the same family. Children up to six years of age often stay with the women around the houses. Children living in valley, most of whom attend school, are independent from a very early age and travel several kilometers a day from the age of six. The youngest boys help to keep the zebus in the meadows near the settlements and, by the time they are 10 to 12 years old, they accompany their fathers to the more distant pastures. Young girls share farming and cooking activities with their mothers and older girls look after the younger children in the absence of adults. Previous research within this society shows that children’s representations of nature include the use of many wild species, a representation that contrasts with adult’s main activity as farmers [37, 38].

For the Betsileo, the lean season (*havaratra*), occurring from mid-October to March just before the rice-harvesting season (*asotry*), is central in structuring livelihood activities. Unlike the rest of the island, where there are two rice harvests, the colder climatic regime of the Namoly valley only allows for one annual harvest of rice, which makes people of the valley more vulnerable to climatic variability. People manage a wide diversity of crops, which traditionally allowed them to cope with variability in rice yield and limited the risk of food shortages [51], although recent change in local climatic patterns harm rice yield and thus extend the lean season [52]. Mainly collected as condiments, side dishes, or for snacking, wild edible plants are also largely used by Betsileo people as a staple food during the lean season.

### Data collection

Fieldwork was conducted in the municipality of Namoly in the district of Ambalavao from January to March 2020 during the lean season, when smallholder farmers are more vulnerable to food crisis, a situation that might increase their reliance on wild edible plants [4, 5, 53]. Before starting data collection, we obtained the agreement from the relevant administrative territorial organizations (*Fokontany* chefs). Free Prior and Informed Consent (FPIC) was requested from each informant before conducting interviews. For children, we requested both their own and their parents FPIC, always reiterating to the child their right to withdraw at any time [54]. This research project was approved by the ethics committee of the Autonomous University of Barcelona CEEAH (4902) and was carried out following the ethical charter of Ethical Research Involving Children [50].

### Sample

Our sample includes women and men and girls and boys. We conducted 82 free listing interviews to collect wild edible data among adults (n = 42) and children (n = 40). We used a convenient sampling method to obtain a balanced sample considering gender and life stage. Our sample includes 22 women, 20 men, 22 girls and 18 boys from 52 households. We also selected eight key informants, five adults and three children, to collect additional data with semi-structured interviews [55]. The five adults (two women and three men) were *ombiasa* (traditional healers working with plants and animals), well-known in the valley for their expertise. As children’s key informants, we selected two boys and one girl around 10 and 12 years old able to cite and identify more than 20 plant species in our free listing exercise (which represents twice the average number of plants cited by children). The eight key informants were not related through kinship.

### Ethnobotanical data

Ethnobotanical data were collected using free listings and semi-structured interviews with key informants. We collected ethnobotanical data using free listings because this tool gives a good overview of local knowledge and can be adapted to children, so data collected using free listings allow us to compare adults’ and children’s knowledge [18].

Because people of the study area do not have a unique term for the concept “wild edible plants’’, our prompt for free listing was: “Which non-cultivated plants do you know that are *edible*?” We used the word *sakafo*, which means food, meal or edible. To facilitate the work with children and to build a relationship of trust with them, before starting data collection we spent two weeks of direct and participant observation [56, 57]. To avoid that children identified the interview with schoolwork, free listing interviews with children were mostly conducted outside school, mostly at home [58, 59]. If parents wanted to assist in the interview, we asked them not to influence children’s responses [58]. Free listing interviews with adults were conducted at home or in communal spaces, like the marketplace.

To obtain more detailed data on each WEP listed in free listing, we conducted semi-structured interviews with adults and children key informants. During semi-structured interviews with key informants, and for each WEPs reported in free listing, we asked about i) synonymy, ii) parts consumed, and iii) wild edible plant location and hamlets-to-location walking time. To collect this information with children, we complemented our semi-structured interview with walks in the surrounding forests. Semi-structured interviews with adult key informants were longer than semi-structured interviews with children (2 hours vs. 45 minutes). Semi-structured interviews with adults were conducted at their home or during wild edible plant collection walks.

### Botanical data

In February 2019, with the help of local experts, we collected and made vouchers for WEP reported during free listings. We prepared plant vouchers following the field guide used by Missouri Botanical Garden [60]. WEPs were identified by comparing the local names, vouchers and photographs taken by the first author with species identified in literature and Malagasy flora database [61–63]. Family and scientific names were checked with the World Flora Online [64]. For each species reported during free listing, we determined its life form. We considered "trees" as plants erected from the ground with a single trunk, "shrubs" as multiple woody stems plants, "climbers" as support-dependent plants, and "herbaceous" as annuals plants without a trunk, usually non-woody. Based on the type of vegetation and disturbance levels on the area [65, 66] (see S1 Table in S1 File for habitats description), we described five ecological habitats where WEP grow: Grassy Secondary Vegetation, Shrubby Secondary Vegetation, Mountain Forest and Highland vegetation. We used the Tropicos database [33] to determine the biogeographical distribution (endemic, native and introduced) of plants in free listings.

### Data analysis

We started the analysis using a MANOVA to test the relation between the habitats and the biogeographical characteristic of the WEPs cited. This test allows us to assess the number of endemic (to Madagascar), native (to Africa including Madagascar and the Mascareignes region) and introduced (voluntarily or accidentally) species in each habitat.

Then, to assess the level to which WEPs knowledge varies within people in the study site, we used gender (i.e., women vs. men), life stage (i.e., adults vs children), and the combination of both to build different subsamples for the analysis.

### Number of WEPs cited and estimated

To get an overview of the WEP knowledge distribution, we used free listing data to calculate the total number of WEP cited–i.e., observed richness -, differentiating between citations by the full sample and by each subsample. We also analyzed the number of WEPs shared and not shared by the four subsamples. Then, we estimated the theoretical number of WEP known–i.e., the estimated richness–using the non-parametric estimator Chao 2, i.e., the asymptote of the accumulation curve of species cited [67]. Estimated richness was calculated for the full sample and for subsamples and compared to the respective observed richness.

To assess variation on the number of wild edible plants listed across gender and life stage, we compared the length of the lists generated by the four subsamples (i.e., women, men, girls and boys) using a Kruskal-Wallis rank sum test. We also used a Welch two sample t-test to compare the mean list length of adults vs. children and of females vs. males. Finally, we investigated the relation of list length with age (discrete variable), gender, and their interaction using a Poisson generalized linear model (GLM) with a log link function.

### Dissimilarity and species’ composition

To explore differences in the WEPs reported associated to the informant’s gender and life stage, we performed an analysis of variance using distance matrices. We started using the Jaccard index to build a dissimilarity matrix of informants vs. species. We then tested for differences between groups using a PERMANOVA, from the ‘*adonis*’ function in the *vegan* package of R [68].

### WEPs importance

To evaluate the influence of gender and life stage on the importance attributed to wild edible plants by each of the subsamples, for each subsample we calculated a cognitive salient index: the *B*’score index [69].

### Wild edible plants characteristics

We described WEPs characteristics according to their life form, parts consumed, growth habitats, and biogeography (see S1 Table in S1 File for habitat variables description). To test whether there are differences in the characteristics of the wild edible plants cited by the four subsamples, we used a MANOVA. Four models were performed including as explanatory variables life stage, gender, and the interaction between them: i) the life form model with four response categories (i.e., herbaceous, trees, shrubs and climbers); ii) the habitat model with five response categories (i.e., HV, MF, SSV, GSV and WET); iii) the part consumed model with seven response categories (i.e., whole, fruit, leaves, new shoots, tuber, seeds, and nectar); and iv) the biogeography model with three response categories (i.e., endemic, native and introduced). The MANOVA tests were performed in R [70] with the package *stats* version 4.0.3. We used a boxplot representation to display the trends elicited by the MANOVA. We also used Kruskal-Wallis chi-squared test as post-hoc to solve the effects of combined explicative variables resulting from MANOVA. Finally, we used a Chi-square test to compare the distribution of endemic, native and introduced species among the ten most salient species–base on the *B*’score index—listed by the four subsamples.

## Results

**Fig 2:**
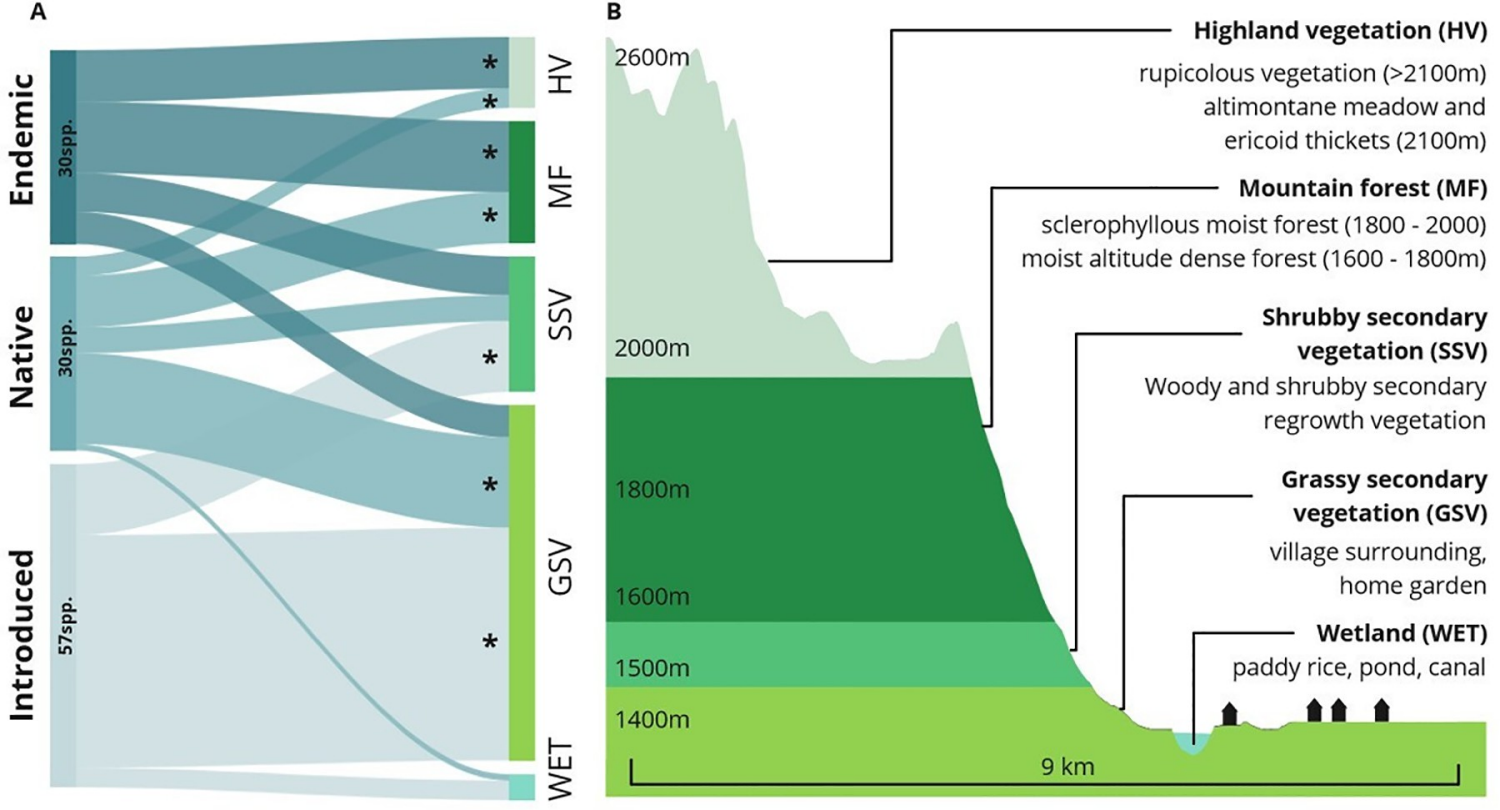
Biogeography and habitat of Betsileo WEPs collection. A. Sankey diagram showing the proportion of endemic, native and introduced WEP species found in each habitat. The thickness of the lines refers to the number of species. “*” indicate significant relation based on MANOVA (threshold at P < 0.001) (S2 Table in S1 File). B. altitudinal zonation of vegetation in Namoly valley from west to east. X and Y axes not at the same scale.

Informant listed a total of 136 local names of wild edibles. Once synonyms were solved, the cleaned list included 117 wild edible plants, one fungus, and two arthropods species. We removed the fungi and the arthropods for the analysis, so our final list includes 117 WEP species, of which we were not able to identify nine plants (S4 Table in S1 File). The plant species reported belong to 52 botanical families and 91 genera. Most of the WEP species reported were herbaceous (n = 57 species), followed by trees (n = 27), shrubs (n = 19) and climbers (n = 14). Many of the WEP cited have multiple parts consumed with different preparation modes (e.g., raw, boiled, braised). For almost half of the species (n = 53), fruits are consumed, usually eaten raw as snacks. Other parts consumed include the whole aerial part (29 species), the tubers (14 species), the leaves (13 species), the new shoots (4 species), the nectar (2 species), and the seeds (2 species). Almost a third of the WEP cited are collected in areas dominated by grassy secondary vegetation (62 species). Fewer WEP are collected in areas dominated by shrubby secondary vegetation (21 species), mountain forest (19 species), highland vegetation (11 species), or wetlands (4 species). About half of the WEP species cited were introduced (57 species), the rest of the species cited were equally distributed between endemic (30 species) and native (30 species). There is a direct relation between the number of introduced, endemic and native’s species of WEPs and their corresponding ecological habitats (S2 Table in S1 File). The richest habitats in endemic species were the habitats with highest altitude and less accessible from settlements in the bottom of the valley (Fig 2).

### Number of WEPs cited by subsamples

The number of WEP species cited by informants did not reach the estimated richness (69.5% of completeness) according to the asymptotic value from Chao’s estimator (S1a Fig in S1 File). Children cited 84 species, representing 68% of the estimated richness, and adults cited 91 species, or 55% of the estimated richness, which suggests that both subsamples might know more species than those cited during our free listings (S1c and S1b Fig in S1 File). Similarly, females cited 97 species, 62% of the estimated richness, and males 85 species, 70% of the estimated richness, which again suggests that both subsamples might know almost a third WEP species more than they cited (S1d and S1e Fig in S1 File).

**Fig 3:**
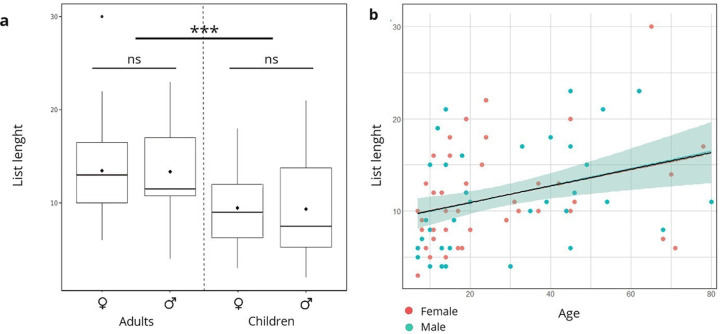
Gender and life stage effect on the number of WEPs cited: a. comparison of the mean WEP species richness cited by subsamples of informants, b. linear correlation between WEP list length and age (female = red dots and male = blue dots). ns = non-significant.

Adults cited more WEP species (avg = 13.40; SD = 5.51) than children (avg = 9.58; SD = 4.71), while female (avg. = 11.45; SD = 5.34) and male (avg. = 11.70; SD = 5.67) respondents cited about the same number of WEP species (Fig 3A). We found that the differences in lists length were statistically significant for life stage (t = 3.3571, df = 78.462, p <0.001) and statistically not significant for gender (t = -0.201, df = 74.869, p = 0.840).

Results from the linear regression of list length against informants’ age show a positive relation between the two variables (estimate = 0.007, Std. error = 0.001 z value = 4.652, p ≤ 0.001***) (Fig 3B). In this same regression, the variable that capture the gender of the informant was statistically insignificant (p = 0.97).

### WEP species’ composition by subsamples

**Fig 4:**
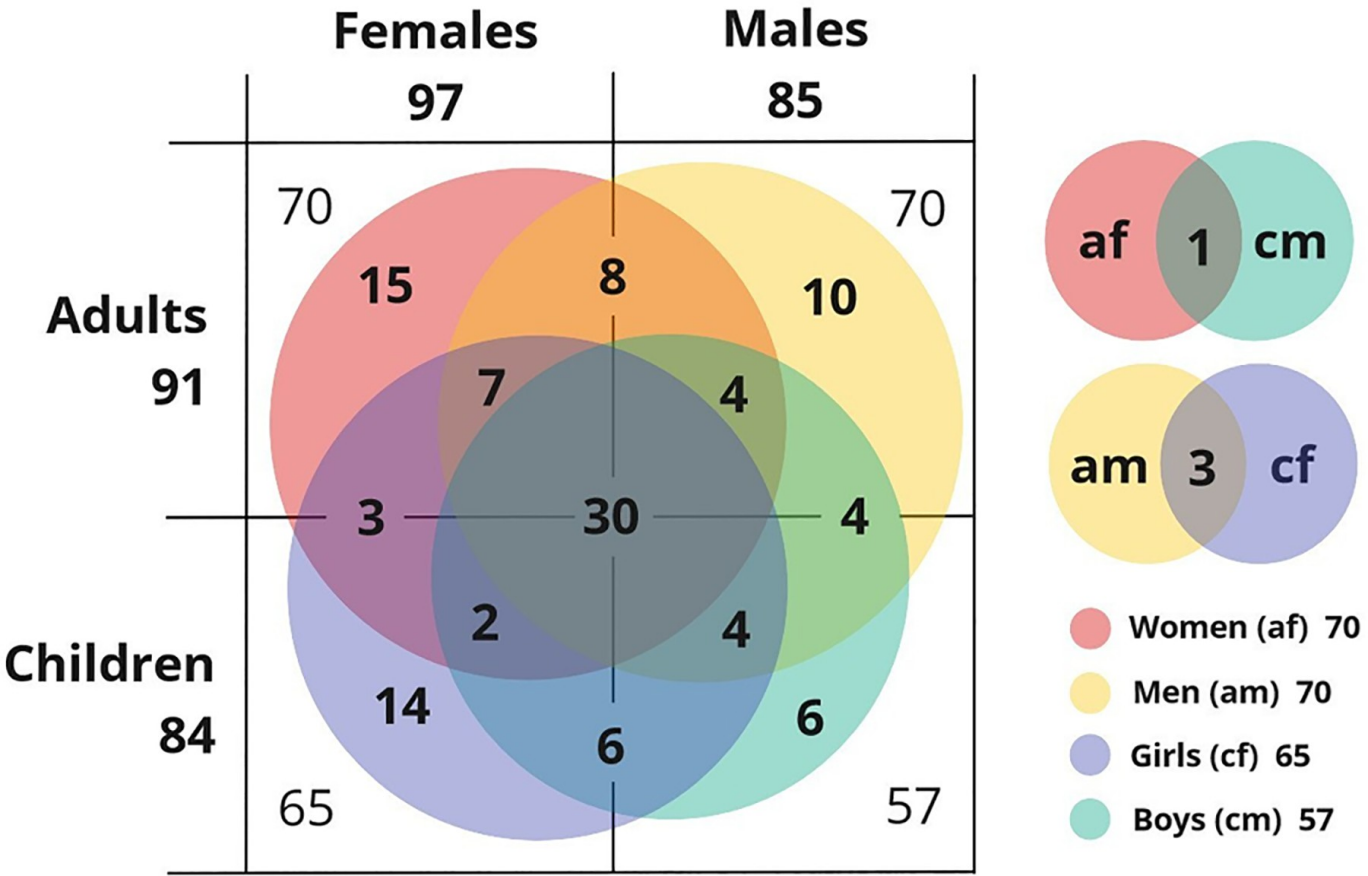
Venn diagram of WEP species shared by the four subsamples. Representation of the number and the distribution of WEP species cited by each subsample.

Only 25% (n = 30 spp.) of the 117 WEP in our list were cited by all subsamples. Most WEP species cited by a subsample were specific to it, i.e., not or rarely cited by other subsamples. Twenty-six WEPs were mentioned only by children and 33 WEPs were only mentioned by adults. Females cited 32 WEP not mentioned by males, while males cited 20 species not listed by females. Most WEP cited only by one of the subsamples were cited only once, and only a few of these species were cited more than five times (Fig 4).

The WEP reported by the different subsamples differs widely. According to results of a PERMANOVA, the WEP listed by adults and children (F = 4.015, R^2^ = 0.047, p = 0.001) and by females and males (F = 1.723, R^2^ = 0.020, p<0.01) are significantly different. Women and men listed different WEP (F = 1.849, R2 = 0.044, p<0.01), but there was no statistically significant difference in the lists of girls and boys (F = 0.875, R^2^ = 0.023, p = 0.672). We also found significant differences between the lists of girls and women (F = 2.568, R^2^ = 0.057, p<0.001), boys and women (F = 1.879, R^2^ = 0.048, p<0.01), girls and men (F = 3.842, R^2^ = 0.087, p<0.001) and boys and men (F = 2.446, R^2^ = 0.065, p<0.001).

### Importance attributed to WEPs

The 30 WEP’s species common to all subsamples are also the most frequently cited within each subsample. According to the *B*’score index, only three WEP species overlap among the ten most salient species in each subgroup (*Aphloia theiformis* (Vahl) Benn. Aphloiaceae, *Syzygium cumini* (L.) Skeels Myrtaceae and *Rorippa laurentii* Jonsell Brassicaceae) (Table 1). *Rorippa laurentii* seems particularly salient for children as, on average, it was listed second in both girls and boys subsamples.

**Table 1:** Ten most salient WEPs species according to the *B*’score index.

	Local name	Scientific name	Family	Biogeography	Habitat	Frequency	*B*’score
	Women						
1	*Lamoty*	*Flacourtia indica* (Burm.f.) Merr.	Salicaceae	N	MF	0.73	0.602
2	*Trakavola*	*Bidens pilosa* L.	Compositae	N	GSV	0.77	0.59
3	*Voafotsy*	*Aphloia theiformis* (Vahl) Benn.	Apholiaceae	N	MF	0.68	0.584
4	*Tsipoaka*	*Rorippa laurentii* Jonsell	Brassicaceae	E	HV	0.68	0.56
5	*Rotsy*	*Syzygium cumini* (L.) Skeels	Myrtaceae	I	SSV	0.64	0.549
6	*Voanaka*	*Physalis peruviana* L.	Solanaceae	I	GSV	0.59	0.425
7	*Kimaosy*	*Vigna angivensis* Baker	Leguminosae	E	GSV	0.55	0.392
8	*Giranadela *	*Passiflora edulis* Sims	Passifloraceae	I	GSV	0.41	0.344
9	*Ovy ala*	*Dioscorea trichantha* Baker	Dioscoreaceae	E	SSV	0.41	0.304
10	*Lanary*	*Plagioscyphus jumellei* (Choux) Capuron	Sapindaceae	E	MF	0.36	0.291
	Men						
1	*Giranadela*	*Passiflora edulis* Sims	Passifloraceae	I	GSV	0.75	0.529
2	*Rotsy*	*Syzygium cumini* (L.) Skeels	Myrtaceae	I	SSV	0.6	0.489
3	*Lamoty*	*Flacourtia indica* (Burm.f.) Merr.	Salicaceae	N	MF	0.55	0.46
4	*Tsipoaka*	*Rorippa laurentii* Jonsell	Brassicaceae	E	HV	0.65	0.456
5	*Voafotsy*	*Aphloia theiformis* (Vahl) Benn.	Apholiaceae	N	MF	0.5	0.431
6	*Voatsitakajaza*	*Vaccinium secundiflorum* Hook.	Ericaceae	E	HV	0.55	0.423
7	*Lanary*	*Plagioscyphus jumellei* (Choux) Capuron	Sapindaceae	E	MF	0.5	0.419
8	*Ovy ala*	*Dioscorea trichantha* Baker	Dioscoreaceae	E	SSV	0.6	0.406
9	*Kilenga*	*Bryophyllum campanulatum* (Baker) (*)	Crassulaceae	E	GSV	0.45	0.294
10	*Kitonda*	*Medinilla* sp.	Melastomataceae	E	MF	0.4	0.294
	Girls						
1	*Voanaka*	*Physalis peruviana* L.	Solanaceae	I	GSV	0.55	0.414
2	*Tsipoaka*	*Rorippa laurentii* Jonsell	Brassicaceae	E	HV	0.59	0.403
3	*Voafotsy*	*Aphloia theiformis* (Vahl) Benn.	Apholiaceae	N	MF	0.45	0.396
4	*Kilela*	*Passiflora subpeltata* Ortega	Passifloraceae	I	GSV	0.55	0.387
5	*Trakavola*	*Bidens pilosa* L.	Compositae	N	GSV	0.5	0.364
6	*Voaroy*	*Rubus rosifolius* Sm.	Rosaceae	I	SSV	0.5	0.349
7	*Goavy*	*Psidium guajava* L.	Myrtaceae	I	SSV	0.41	0.318
8	*Rotsy*	*Syzygium cumini* (L.) Skeels	Myrtaceae	I	SSV	0.36	0.293
9	*Roimboza*	*Lantana camara* L.	Verbenaceae	I	GSV	0.41	0.274
10	*Ana*	*Solanum* cf. *nigrum*	Solanaceae	I	GSV	0.32	0.214
	Boys						
1	*Goavy*	*Psidium guajava* L.	Myrtaceae	I	SSV	0.5	0.371
2	*Tsipoaka*	*Rorippa laurentii* Jonsell	Brassicaceae	E	HV	0.5	0.329
3	*Voafotsy*	*Aphloia theiformis* (Vahl) Benn.	Apholiaceae	N	MF	0.39	0.308
4	*Lamoty*	*Flacourtia indica* (Burm.f.) Merr.	Salicaceae	N	MF	0.33	0.295
5	*Paiso*	*Prunus persica* (L.) Batsch	Rosaceae	I	GSV	0.33	0.29
6	*Rotsy*	*Syzygium cumini* (L.) Skeels	Myrtaceae	I	SSV	0.33	0.278
7	*Kilela*	*Passiflora subpeltata* Ortega	Passifloraceae	I	GSV	0.39	0.268
8	*Voanaka*	*Physalis peruviana* L.	Solanaceae	I	GSV	0.39	0.252
9	*Roimboza*	*Lantana camara* L.	Verbenaceae	I	GSV	0.33	0.252
10	*Trakavola*	*Bidens pilosa* L.	Compositae	N	GSV	0.39	0.226

Biogeography: (E: endemic to Madagascar, N: native to Africa including Madagascar and the Mascareignes region I: introduced in Madagascar). Habitat: MF (mountain forest), HV (highland vegetation), SSV (shrubby secondary vegetation) and GSV (grassy secondary vegetation). (*) *Bryophyllum campanulatum* (Baker) V.V.Byalt, Udalova & I.M.Vassiljeva.

### Wild edible plants characteristics

**Fig 5:**
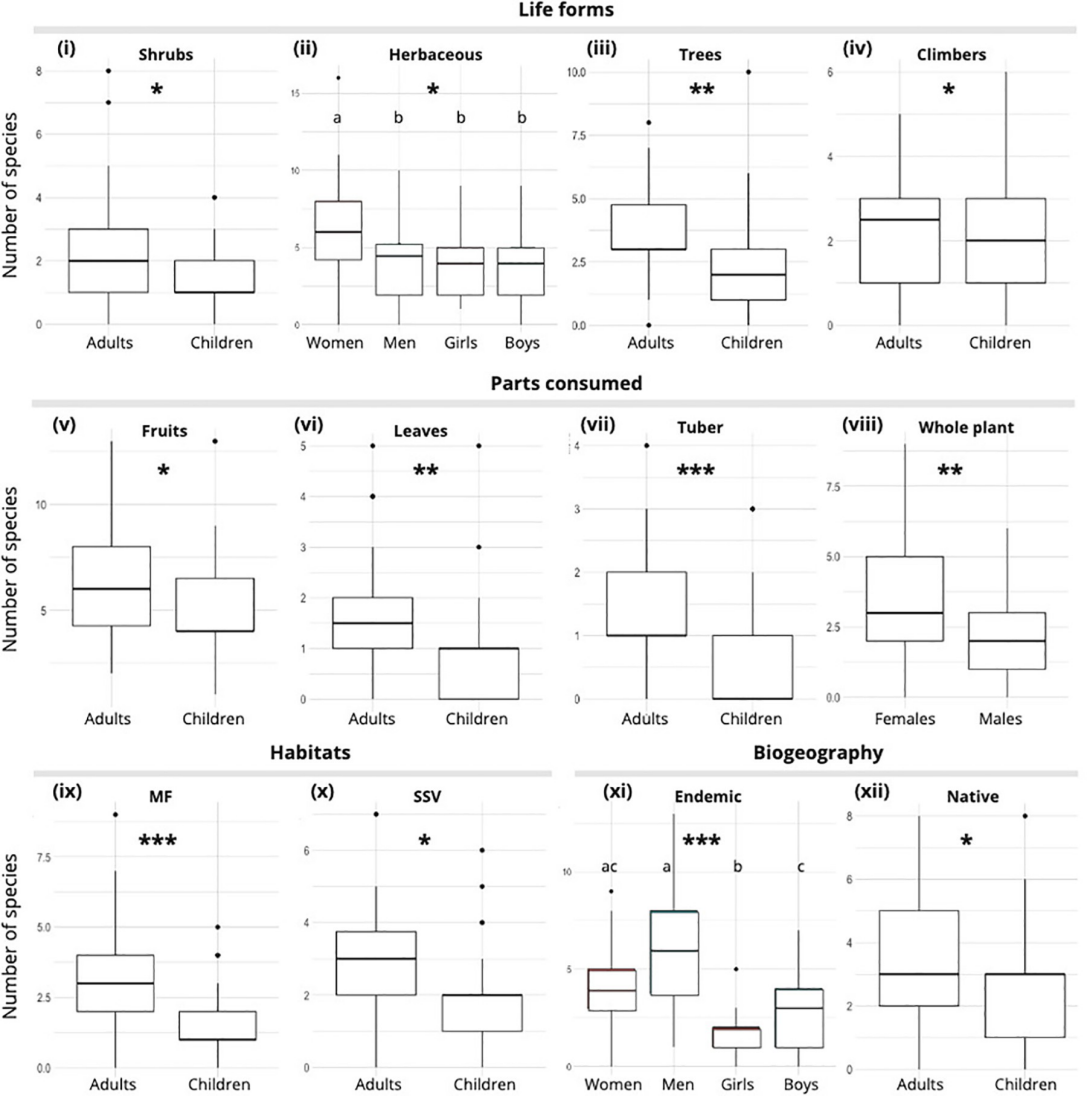
Number of WEPs cited by categories and subsamples. “*” indicate significant relations based on MANOVA. Letters indicate a similar number of plant species cited based on Kruskal-Wallis chi-squared (threshold at P < 0.01).

The subsamples of adults and children and, to some extent, women and men, listed WEP with different life forms, parts consumed, ecological habitats, and biogeographical distributions (S3 Table in S1 File, Fig 5). Results from a MANOVA test suggest significant differences in the number of shrubs, trees, and climber’s species cited by adults and children, with adults citing more WEP in these life forms than children (S3 Table in S1 File, Fig 5i, 5iii and 5iv). The subsample of women significantly cited more herbaceous WEPs than any other subsample (S3 Table in S1 File, Fig 5ii).

A similar analysis suggested that WEPs eaten for their fruits, leaves, or tubers were more cited by adults than by children (Fig 5v, 5vi and 5vii). WEPs eaten as whole were significantly more cited by women and girls than by men and boys (Fig 5viii).

Regarding ecological habitat, adults cited more WEPs species from mountain forest habitat (p-value < 0.001) and from shrubby secondary vegetation (p-value < 0.05) than children (S3 Table in S1 File, Fig 5ix and 5x). We did not find a statistically significant difference between the number of WEP species from grassy secondary vegetation cited by adults and children, suggesting that the two subsamples listed about the same number of WEPs from this ecological habitat. We did not find any gender difference on the number of WEP cited for any habitats.

Regarding biogeographical distribution of WEP, we did not find differences across subsamples in the number of introduced WEP species listed. However, we found that adults cited more endemic and native WEP species than children (S3 Table in S1 File, Fig 5xi and 5xii). It is worth noting that men cited a significantly higher number of WEP endemic species than other informant’s subgroups (Fig 5xi).

**Fig 6:**
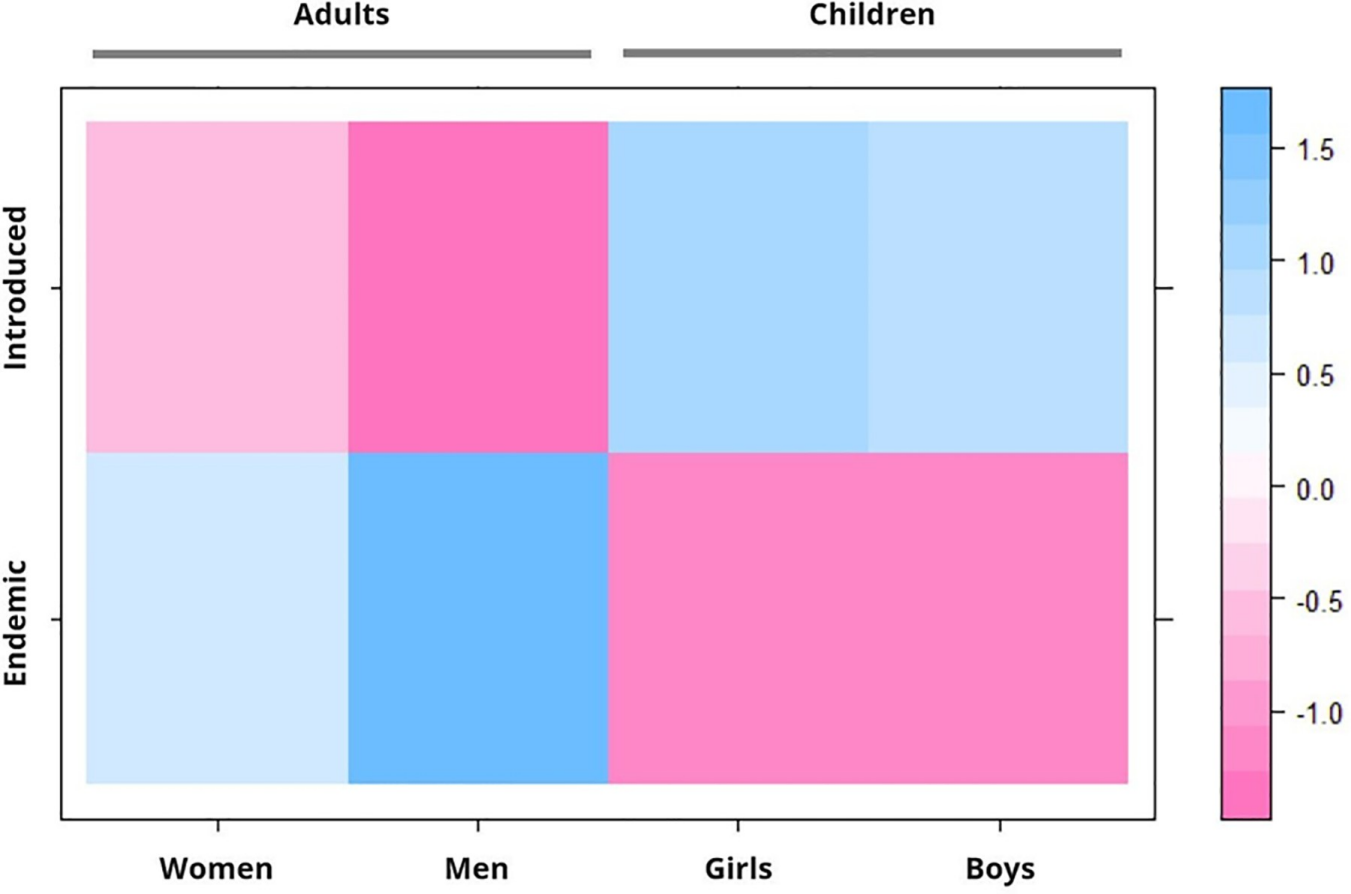
Pearson’s residuals from a chi-square test on biogeographical characteristics of WEPs cited by informants using subsample based on *B*’score rank.

Finally, we found that the distribution of endemic and introduced species in the list of the ten most salient species cited by adult and children was significantly different from what would be expected if the distribution was random (Chi-square = 9.389, df = 3, p-value = 0.024). Indeed, according to Pearson’s residuals value from a chi-square test (Fig 6), in their respective lists, adults cited more endemic species as salient than children, while children cited more introduced species as salient than adults.

## Discussion

Before discussing our results, we want to acknowledge that we are aware that results presented might be biased. A main source of bias results from the use of free listing for data collection. The free listing method calls upon the memory thus some information might have been lost during interviews [71, 72]. In that sense, we acknowledge that the knowledge on WEP presented here might not be exhaustive, indeed our analysis shows that the number of species mentioned by the participants did not reach the estimated richness (S1 Fig in S1 File). Despite this caveat, our converging results show an important intracultural variation in WEP’s knowledge distribution.

### Intracultural variation of knowledge

Wild edible plants knowledge (i.e., number of WEP listed, the species listed, and their botanical characteristics) is differentially distributed among the Betsileo population. While sharing knowledge on a common set of WEP species, Betsileo women, men, girls and boys from Namoly valley have differentiated knowledge for most WEP species. Indeed, the small proportion of WEP known by all subgroups (i.e., 25% of all species listed) suggests that the WEP knowledge system is heterogeneously distributed.

Previous work has shown that sharing a large “core set” of knowledge in an extreme situation of plant scarcity and in an unpredictable environment can enhance group’s resilience and adaptation strategies [73, 74]. However, having differentiated knowledge within the same group might be also beneficial, as it enlarges the set of useful species to rely on [75]. Indeed, in the same way a high plant diversity is essential to maintain nature’s contributions to people [76]. Relying on a large and diverse set of useful plant species allows overlap in the knowledge system regarding species with similar functions and supporting similar services [77]. Because it enhances utilitarian redundancy, local knowledge on a diversity of plants providing similar services can contribute to enforce the adaptive capacity and resilience of the knowledge system [16, 77].

According to our results, knowledge on WEP differs widely across life stages and gender, with children displaying an extended WEP knowledge of their own. In fact, our results support the argument that the different roles they play in society make that females, males, adults and children relate differently with nature, including individual and social daily experience shape their knowledge [12].

### Life stage and children’s knowledge

Betsileo children participating in this study were able to list a large number of WEP (84 species, 69% of which are in common with adults). This is one of the largest numbers of WEP listed found in research on the topic. In fact, among the ten studies we found reporting the number of WEP known by children, only one reported a higher number of WEP species known by children: a study among mestizo children in the Peruvian Amazon [78].

Our research also shows that children know different WEP species than adults, rank them in a different way, and that WEP species known by children and adults have different characteristics. These differences between children and adults might bring some new insights into our understanding of plant biodiversity. For instance, the fact that children ranked WEP differently than adults raises not previously considered conservation issues. For example, *Rorippa laurentii* Jonsell, a species particularly salient for children, is a rare species known only in the study area [33] and not yet evaluated by the IUCN red list, nor recorded as a useful plant in the world checklist of useful plant species [79]. Given the importance of this species for children, it might work evaluate its ecological status to manage it accordingly.

Differences between children and adults WEP knowledge highlighted by our results might be discussed through the lenses of socio-cultural, ecological and cultural transmission contexts. In particular, we advance three arguments.

First, adults and children have differentiated knowledge because the social organization in which they life frames their mobility and their daily activities and access to diverse habitats and plant species. In the study site, Betsileo children’s mobility is limited to the surrounding settlements. Around the age of 12, Betsileo girls and boys become more independent and go to farther areas with different habitats, like forests where they harvest timber, fuel, or other products. Increasing mobility provides Betsileo children the opportunity to explore new habitats and gather different WEP. Unlike children, adults’, and specially men’s, daily activities (shepherding, hunting, fuel and other NTFP harvest) put them in contact with remote, less disturbed areas, such as ericoid thickets, where high levels of biodiversity and a high rate of endemism remains [46]. Interestingly, we noticed that most WEP growing in habitats close to the settlements were equally known by both adults and children. These results highlight that specific sets of knowledge are acquired very early in life and then maintained through it, while others are learned or transmitted during adulthood due to the apparition of new specific activities and increasing mobility [37].

Second, environmental changes in plant distribution leading to intergenerational knowledge differences might also explain differences between adults and children. Indeed, our results show that adults cited endemic species more frequently and earlier in their lists, whereas the pattern was opposite for children, among whom introduced species were more salient. According to the ecological apparency hypothesis, there is a positive correlation between the cultural salience of a species and its distribution and abundance [80]. In the study area, endemic and introduced species were not equally distributed between ecological habitats, but followed an altitudinal gradient with more introduced species in the bottom of the valley, where people live (Fig 2). Indeed, most introduced species have synanthropic behavior and growth abundantly around human settlements [81] (Fig 2), for which children, mostly living and playing around houses, have daily interactions with introduced species. A complementary explanation to the finding that introduced species were more salient for children than for adults relate to differences in their baselines. A high number of plants species have been introduced to Madagascar during the last two decades [82], for which children might now be exposed to introduced species that adults and elders did not know during childhood for the same ecological habitat. The fact that each generation perceives and interact differently with biodiversity due to environmental changes occurring in the meantime has been used to explain lack of perception of change in younger generations under the idea that there is a “shifting baseline syndrome” [83, 84]. Here, given the rapid land cover change in the highlands of Madagascar [34, 35], the hypothesis of a “shifting baseline syndrome” can be used to explain differences in WEP knowledge between adults and children, where children have differentiated knowledge of new species.

Third, the fact that Betsileo children hold a WEP knowledge that is distinct from that of adults might be also explained by horizontal mechanisms of knowledge transmission and acquisition. As in other small-scale societies [25], Betsileo children learn alongside their parents: however, they spend most of their time with each other. During this time without adults, playing or engaging in subsistence activities, children learn from each other as it has also been observed in other communities [24, 27, 28, 85, 86]. This behavior might support the development of specific language elements and WEP knowledge [24]. A growing corpus of studies leads us to consider horizontal transmission as a predominant way for children to receive knowledge in small-scale societies [18]. Other aspects related to ontogenetic of cultural learning might explain such variation between adults and children [87]. Factors such as the motor/physical and sensorial development during childhood, taking part of an embodied learning [88], should be taken into consideration in further studies, especially the ones focusing on edible plants as organoleptic aspects shape food choices, and thus practices and knowledge related to food [89, 90].

### Gendered differences in WEP knowledge

Our results show that women and men listed about the same number of WEP, although the specific WEP in their lists were different. This finding is not new and have been shown across different societies and knowledge domains including medicinal plants [91, 92], hunting [93], wild edibles [94], and home garden biodiversity [95]. In line with these findings, our results show that women and men listed WEP with different life forms and from which different parts are consumed. For example, girls as women listed more plants eaten as a whole than males; and women listed more herbaceous plants (i.e., leafy vegetables) than men. Most of these plants cited by women and girls, characterized by a similar life forms (herbaceous) and parts consumed (whole aerial part), locally known as *traka*, are central component in many dishes mainly prepared by females. These plants (*traka*) are usually boiled to be eaten with rice as condiment while the broth (*ro*) is drunk and appreciated for is taste [96]. Early in life, girls learn from their mothers how to collect and prepare them, which may explain the important knowledge overlap between girls and women. According to our result, this Betsileo female knowledge seems to be structured around a specific plant life form or type (*traka*)—defined by their accessibility, abundance, morphology and preparation mode (boiled) regardless of the species themselves. The finding is consistent with the idea proposed by Bahuchet [97], that the preparation mode provides a rigid framework for ingredients selection, where ingredients can change but will always be chosen and adapted to the preparation mode.

As for life stages, gendered mobility associated to social organization and daily activities also shapes WEP knowledge. For example, the fact that females in our sample listed more herbaceous plants than men could be explained by the specificity of girl and women mobility due to strong gendered division of labor. Indeed, most herbaceous plants cited by females grow in grassy secondary vegetation, such as home gardens and houses surroundings, where most of female’s activities are performed. Differently, when men take the zebus to remote highland pastures, they pass through many undisturbed ecological habitats (see Fig 2) and thus are exposed to a different plant species, which might explain the high number of endemic plant species known by men in comparison to women.

Finally, our results are innovative in showing that the gendered patterns in knowledge distribution also vary according to the intersectionality of gender and life stage: differences among WEP knowledge are larger between women and men than between girls and boys. Few studies have focused on the knowledge differences between girls and boys, but the few existing studies on the topic show that knowledge about WEP is similar among girls and boys [24, 86]. Girls and boys growing together under an alloparental scheme and in the same domestic environment share a common set of knowledge [24, 86]. The gradual gendered differentiation in knowledge is due to the progressive incorporation of normative rules of the society as children age [98] and to the progressive gendered division in daily activities, from socialization and to division of labor [25].

## Conclusion

Our results show that wild edible plant knowledge is differentially distributed among the Betsileo population in the study site. We provide solid evidence that life stage and gender are determinants in this intracultural variability and generate specific sets of knowledge across different groups. Certain sets of WEP knowledge are acquired during adulthood while other specific sets of knowledge are acquired very early, making children reliable knowledge holders.

In addition, over the lifetime, gender plays an important part in the specialization of WEP knowledge. Our study stresses the need to explore more the complexities of intersectionality in knowledge acquisition to catch intracultural variations of knowledge in small-scale societies as exhaustively as possible by emphasizing children. It also suggests that embedding children into further studies on LEK might contribute to our understanding of biodiversity and raise conservation issues. Given our findings and the growing literature in the emerging field of children ethnoecology, we call for further studies to consider children when exploring local ecological knowledge, and for doing so, to use tools and methodologies adapted to children.

## Supporting information

S1 File (PDF)
