# Supplementary material for: Growing up in the Betsileo landscape: Children’s wild edible plants knowledge in Madagascar
Source: PLoS One. 2022 Feb 17;17(2):e0264147. doi: 10.1371/journal.pone.0264147 (PMC8853535; doi:10.1371/journal.pone.0264147)
Supplement: S1 File — (PDF) [file pone.0264147.s001.pdf]

Supporting information

S1 Table. Habitat variable description.

Shrubby Secondary Vegetation (SSV)	Including a mosaic of secondary regrowth vegetation both woody and shrubby mainly open and colonized by ferns.
Grassy Secondary Vegetation (GSV)	including village surroundings, home garden, pastures and crops fields.
Mountain Forest (MF)	including moist altitude dense forest (1500 - 1800m) and sclerophyllous moist forest (1800 – 2000m).
Highland Vegetation (HV)	including altimontane meadows, ericoid thickets (2100m) and rupicolous vegetation (2500m).
Wetland (WET)	including paddy rice, pond and canal.

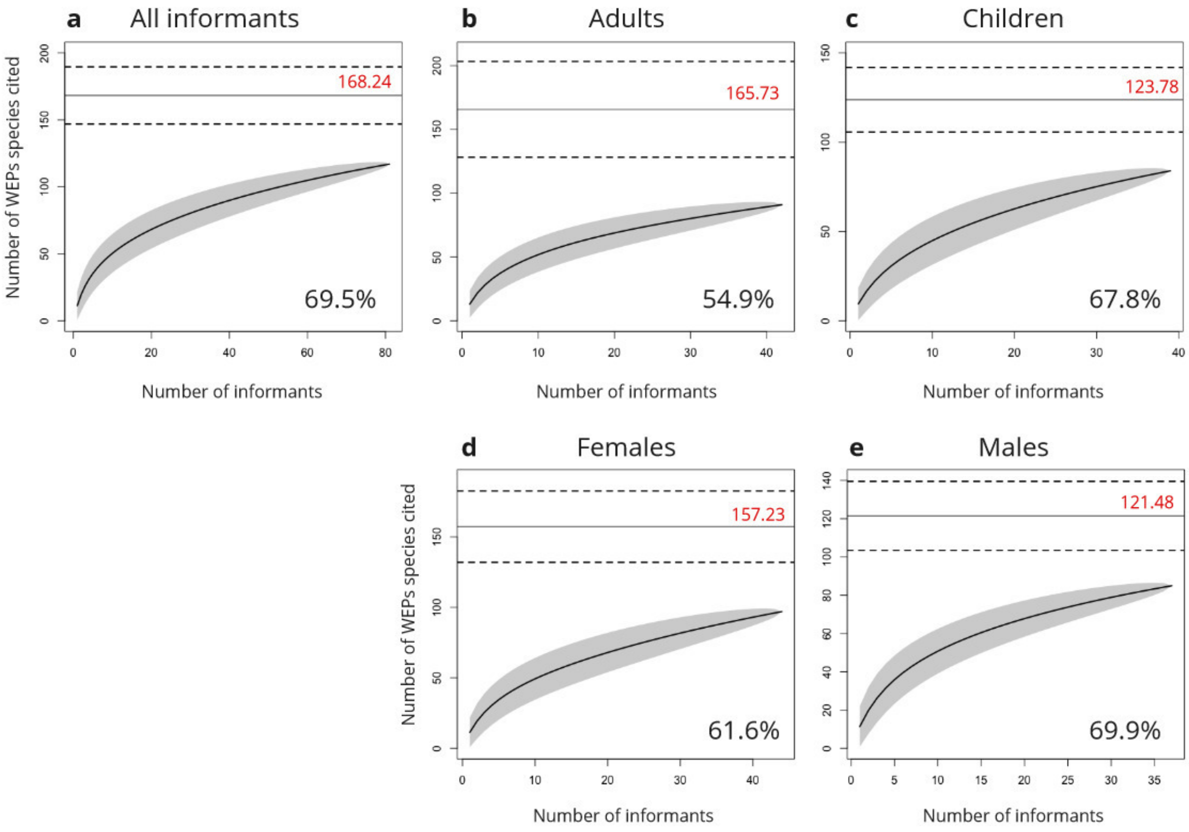

S1 Figure. Accumulation curves of WEPs species and sampling completeness. a. full dataset with all informants, b. adults, c. children, d. females and e. males. The red number indicates the richness expected,

calculated with Chao2 estimator. The percentage indicates the sampling completeness i.e., the ratio between observed and expected richness.

S2 Table. Relation between habitat and the biogeographical characteristic of the WEPs (MANOVA)

Factor	Df	Sum of Squares	Mean Square	F value	p-value
Endemic					
HV	1	147.000	147.000	53.5186	1.266e-09 ***
MF	1	252.039	252.039	91.7607	3.063e-13 ***
SSV	1	24.504	24.504	8.9213	0.004232 **
Introduced					
HV	1	62.946	62.946	29.2018	1.509e-06 ***
SSV	1	125.000	125.000	57.9899	4.135e-10 ***
GSV	1	180.348	180.348	83.6663	1.456e-12 ***
Native					
HV	1	46.667	46.667	24.8868	6.672e-06 ***
MF	1	66.255	66.255	35.3328	2.096e-07 ***
SSV	1	10.843	10.843	5.7827	0.01964 *
GSV	1	45.100	45.100	24.0515	8.989e-06 ***

S3 Table. Multivariate analysis of variance (MANOVA) of characteristics of the WEPs cited with gender and life stage of informants. MF=mountain forest, SSV=shrubby anthropogenic vegetation.

Variables	Factors	Df	Sum of Squares	Mean Square	F value	p-value
**Plant life forms**						
Shrubs	life stage	1	9.539	9.5391	4.5735	0.03564 *
	gender	1	2.465	2.4650	1.1819	0.28037
	life stage*gender	1	6.952	6.9517	3.3330	0.07178 .

Herbaceous	life stage	1	33.33	33.333	4.5521	0.03606 *
	gender	1	25.51	25.514	3.4842	0.06576 .
	life stage*gender	1	30.60	30.600	4.1788	0.04435 *
Tree	life stage	1	28.484	28.4835	9.4054	0.002984 **
	gender	1	4.066	4.0663	1.3427	0.250136
	life stage*gender	1	0.262	0.2617	0.0864	0.769593
Climbers	life stage	1	7.704	7.7038	4.5352	0.0364 *
	gender	1	1.818	1.8183	1.0704	0.3041
	life stage*gender	1	2.670	2.6696	1.5716	0.2138
**Part consumed**						
Fruit	life stage	1	46.73	46.730	6.0180	0.01642 *
	gender	1	10.77	10.768	1.3868	0.24258
	life stage*gender	1	9.50	9.501	1.2235	0.27211
Leaves	life stage	1	13.493	13.4931	9.6538	0.002646 **
	gender	1	0.119	0.1185	0.0848	0.771686
	life stage*gender	1	2.420	2.4197	1.7312	0.192165
Tuber	life stage	1	15.176	15.1763	15.6736	0.0001665 ***
	gender	1	2.994	2.9940	3.0921	0.0826481 .
	life stage*gender	1	0.260	0.2604	0.2690	0.6055089
Whole	life stage	1	3.651	3.6511	1.2407	0.26880
	gender	1	28.855	28.8545	9.8055	0.00246 **
	life stage*gender	1	5.599	5.5989	1.9026	0.17178
New shoots	life stage	1	0.2692	0.269231	1.7891	0.1850
	gender	1	0.0315	0.031487	0.2092	0.6487
	life stage*gender	1	0.1118	0.111849	0.7432	0.3913
Seeds	life stage	1	0.0043	0.004341	0.0369	0.8482
	gender	1	0.1921	0.192108	1.6327	0.2052
	life stage*gender	1	0.2496	0.249563	2.1210	0.1494
Nectar	life stage	1	0.0570	0.057048	0.8097	0.37102
	gender	1	0.0737	0.073704	1.0461	0.30961
	life stage*gender	1	0.2466	0.246583	3.4998	0.06518
**Habitats**						
MF	life stage	1	73.369	73.369	30.5634	4.25e-07 ***
	gender	1	2.857	2.857	1.1901	0.2787
	life stage*gender	1	0.587	0.587	0.2447	0.6223
SSV	life stage	1	11.795	11.7955	5.8688	0.01776 *
	gender	1	2.220	2.2198	1.1044	0.29658
	life stage*gender	1	0.113	0.1135	0.0564	0.81283
GSV	life stage	1	2.55	2.5530	0.3063	0.5816
	gender	1	14.50	14.4958	1.7390	0.1912
	life stage*gender	1	6.52	6.5232	0.7825	0.3791
HV	life stage	1	1.292	1.2918	1.1067	0.29610
	gender	1	0.880	0.8797	0.7536	0.38803
	life stage*gender	1	3.502	3.5022	3.0003	0.08725 .
WET	life stage	1	0.0299	0.029915	0.1891	0.6648

	gender	1	0.0131	0.013106	0.0829	0.7742
	life stage*gender	1	0.0009	0.000859	0.0054	0.9414
**Biogeography**						
Endemic	life stage	1	166.56	166.564	30.3341	4.626e-07 ***
	gender	1	33.45	33.452	6.0922	0.0158 *
	life stage*gender	1	1.79	1.794	0.3268	0.5692
Native	life stage	1	17.026	17.0263	4.5473	0.03616 *
	gender	1	2.027	2.0268	0.5413	0.46413
	life stage*gender	1	1.528	1.5278	0.4080	0.52486
Introduced	life stage	1	1.18	1.1819	0.1822	0.6707
	gender	1	10.81	10.8114	1.6664	0.2006
	life stage*gender	1	0.01	0.0055	0.0008	0.9768

S4 Table. Wild edible plant list (n=117)

Betsileo	Scientific name	Family
Kisonjosonjo	*Sagittaria guayanensis* Kunth	Alismataceae
Tsipotika	*Achyranthes aspera* L.	Amaranthaceae
Komohay la	*Amaranthus blitum* L.	Amaranthaceae
Komohay favy	*Amaranthus graecizans* L.	Amaranthaceae
Anahazo	*Amaranthus retroflexus* L.	Amaranthaceae
Komohay	*Amaranthus spinosus* L.	Amaranthaceae
Sakoa	*Sclerocarya birrea* (A.Rich.) Hochst.	Anacardiaceae
Voafotsy	*Aphloia theiformis* (Vahl) Benn.	Aphloiaceae
Matanandro	*Centella asiatica* (L.) Urb.	Apiaceae
Kifoky	*Cynanchum sessiliflorum* (Decne.) Liede	Apocynaceae
Taro	*Colocasia esculenta* (L.) Schott	Araceae

Via	*Typhonodorum lindleyanum* Schott	Araceae
Bokony	*Polyscias carolorum* Bernardi	Araliaceae
Varobe	*Aloe conifera* H.Perrier	Asphodelaceae
Komotodoha	*Acmella caulirhiza* Delile	Asteraceae
Trakavola	*Bidens pilosa* L.	Asteraceae
Ananambo	*Crassocephalum crepidioides* (Benth.) S.Moore	Asteraceae
Ambiaty	*Gymnanthemum appendiculatum* (Less.) H.Rob.	Asteraceae
Vihia	*Mikania microptera* DC.	Asteraceae
Kilongo	*Senecio erechtitioides* Baker	Asteraceae
Tohiravy	*Phyllarthron cf bojeranum*	Bignogniaceae
Anatsonga	*Brassica juncea* (L.) Czern.	Brassicaceae
Anadranodia	*Nasturtium officinale* W.T.Aiton	Brassicaceae
Anapisaka	*Rorippa insularis* Jonsell	Brassicaceae
Tsipoaka	*Rorippa laurentii* Jonsell	Brassicaceae
Anana androbosy	*Rorippa millefolia* (Baker) Jonsell	Brassicaceae
Raketa	*Opuntia monacantha* (Willd.) Haw.	Cactaceae
Voakitaitay	*Rhipsalis baccifera* (J.S.Muell.) Stearn	Cactaceae

Voasipikopiko	*Canna orchiodes* L.H.Bailey	Cannaceae
Fandrindambo	*Cadaba virgata* Bojer	Capparaceae
Kimba	*Symphonia gymnoclada* (Planch. & Triana) Benth. & Hook.fil. ex Vesque	Clusiaceae
Kilenga	*Bryophyllum campanulatum* (Baker) V.V.Byalt, Udalova & I.M.Vassiljeva	Crassulaceae
Apanga	*Pteridium aquilinum* (L.) Kuhn	Dennstaedtiaceae
Faskiasy	*Didymeles perrieri* Leandri	Didymelaceae
Ovy be	*Dioscorea esculenta* (Lour.) Burkill	Dioscoreaceae
Ovy ala	*Dioscorea trichantha* Baker	Dioscoreaceae
Tavolo	*Tacca leontopetaloides* (L.) Kuntze	Dioscoreaceae
Voatsitakajaza	*Vaccinium secundiflorum* Hook.	Ericaceae
Menahy	*Erythroxylum nitidulum* Baker	Erythroxylaceae
Kily	*Tamarindus indica* L.	Fabaceae
Kimaosy	*Vigna angivensis* Baker	Fabaceae
Sonjo parakandro	*Hypoxis angustifolia* Lam.	Hypoxidaceae
Kindaindaivola	*Gladiolus dalenii* Van Geel	Iridaceae
Sely	*Grewia repanda* Baker	Malvaceae
Kihasihasi	Unknown 1	Malvaceae

Sitrotroka	*Antherotoma naudinii* Hook.fil.	Melastomataceae
Kitonda	*Medinilla ibityensis* H.Perrier	Melastomataceae
Voafandra	*Medinilla* sp.	Melastomataceae
Toamamy	*Rousseauxia andringitrensis* (H.Perrier) Jacq.-Fél.	Melastomataceae
Tsitsitrotroka	*Tristemma mauritianum* J.F.Gmel.	Melastomataceae
Voandelaka	*Melia azedarach* L.	Meliaceae
Nonoka	*Ficus rubra* Vahl<br>*Ficus reflexa* Thunb.	Moraceae
Torovoka	*Ficus* sp.	Moraceae
Ara	*Ficus tiliifolia* Baker	Moraceae
Fopoho	*Ficus trichoclada* Baker	Moraceae
Aviavy	*Ficus trichopoda* Baker	Moraceae
Voaroihazo	*Morus alba* L.	Moraceae
Goavy tsinahy	*Psidium cattleianum* Afzel. ex Sabine	Myrtaceae
Goavy	*Psidium guajava* L.	Myrtaceae
Rotsy ala	*Syzygium bernieri* (Drake) Labat & G.E. Schatz	Myrtaceae
Rotsy	*Syzygium cumini* (L.) Skeels	Myrtaceae
Kivengy	*Nephrolepis tuberosa* (Bory ex Willd.) C.Presl	Nephrolepidaceae
Singilofo	*Cynorkis speciosa* Ridl.	Orchidaceae

Kisira	*Oxalis corniculata* L.	Oxalidaceae
Kisirakisira	*Oxalis latifolia* Kunth	Oxalidaceae
Kisira pahe	*Oxalis* sp.	Oxalidaceae
Fagnasy	*Oxalis xiphophylla* Baker	Oxalidaceae
Grenadelle	*Passiflora edulis* Sims	Passifloraceae
Grenadelle ala	*Passiflora suberosa* L.	Passifloraceae
Kilela	*Passiflora subpeltata* Ortega	Passifloraceae
Hazotra	*Phyllanthus* cf *iratsiensis* Leandri	Phyllantaceae
Melo hazo	*Phytolacca americana* L.	Phytolaccaceae
Sakavirohazo	*Piper borbonense* (Miq.) C.DC.	Piperaceae
Kilanjy	*Sporobolus subulatus* Hack.	Poaceae
Anapoza	*Portulaca oleracea* L.	Portulacaceae
Kitrembo	*Embelia concinna* Baker	Primulaceae
Voarafy	*Maesa lanceolata* Forssk.	Primulaceae
Tsinefo	*Ziziphus jujuba* Mill.	Rhamnaceae
Pibasy	*Eriobotrya japonica* (Thunb.) Lindl.	Rosaceae
Paiso	*Prunus persica* (L.) Stokes	Rosaceae
Voankazo	*Prunus* sp.	Rosaceae
Petity	*Pyracantha angustifolia* (Franch.) C.K.Schneid.	Rosaceae

Roy roy	*Rubus alceifolius* Poir.	Rosaceae
Voaroifotsy	*Rubus apetalus* Poir.	Rosaceae
Voaroitsaka	*Rubus myrianthus* Baker	Rosaceae
Voaroy	*Rubus rosifolius* Sm.	Rosaceae
Kisanga mena	*Anthospermum emirnense* Baker	Rubiaceae
Fatora	*Bremeria scabrella* (Wernham) A.P.Davis & Razafim.	Rubiaceae
Magnesy	*Chassalia bojeri* Bremek.	Rubiaceae
Voalady	*Psychotria cf isalensis* (Bremek.) A.P.Davis & Govaerts	Rubiaceae
Voasary	*Citrus sinensis* (Mill.) Pers., 1806	Rutaceae
Nakasimba	*Toddalia asiatica* (L.) Lam.	Rutaceae
Lamoty	*Flacourtia indica* (Burm.fil.) Merr.	Salicaceae
Lanary	*Plagioscyphus jumellei* (Choux) Capuron	Sapindaceae
Voamasakatany	*Perrierodendron quartzitorum* J.-F.Leroy, Lowry, Haev., Labat & G.E.Schatz	Sarcolaenaceae
Kinananakoho	*Datura stramonium* L.	Solanaceae
Tsipoapoaka	*Nicandra physalodes* (L.) Gaertn.	Solanaceae
Voanaka	*Physalis peruviana* L.	Solanaceae
Anamafaitra	*Solanum americanum* Mill.	Solanaceae

Voatabiahazo	*Solanum betaceum* Cav.	Solanaceae
Voangivy	*Solanum erythrocarpon* G.Mey.	Solanaceae
Seva	*Solanum mauritianum* Scop.	Solanaceae
Anamamy	*Solanum nigrum* L.	Solanaceae
Anadia	*Solanum scabrum* Mill.	Solanaceae
Ana	*Solanum* sp.	Solanaceae
Voapoala	*Solanum torvum* Sw.	Solanaceae
Roimboza	*Lantana camara* L.	Verbenaceae
Voloboka	*Cissus vitiginea* L.	Vitaceae
Longoza	*Hedychium coronarium* J.Koenig	Zingiberaceae
Alamangisy	Unknown 2	
Anadraza	Unknown 3	
Fomoritany	Unknown 4	
Mamijazay	Unknown 5	
Soamorondrano	Unknown 6	
Trakanala	Unknown 7	
Voadandamboa	Unknown 8	
Voamasatenga	Unknown 9	
